# Sonographic characteristics of local soft tissue recurrence in primary bone tumor and diagnostic efficacy versus MRI

**DOI:** 10.1186/s12885-025-14071-6

**Published:** 2025-04-10

**Authors:** Ping Yu, Junxue Gao, Yue Hu, Jiaan Zhu, Yu Wang

**Affiliations:** 1 Department of Ultrasound, Peking University People’s Hospital, Beijing, 100044 China; 2 Department of Ultrasound, Xiang’an Hospital of Xiamen University, Xiamen, 361101 China

**Keywords:** Ultrasound, Magnetic resonance imaging, Local recurrence, Primary bone tumors, Limb salvage, Logistic regression analysis

## Abstract

**Background:**

The accurate diagnosis of local soft tissue recurrence (LR) in primary bone tumors is crucial for guiding clinical management and predicting patient outcomes. However, standardized postoperative surveillance protocols remain undefined. This study aims to compare the diagnostic efficacy of ultrasound (US) versus magnetic resonance imaging (MRI) in detecting LR following primary bone tumor surgery and to characterize the sonographic features of osteosarcoma recurrence.

**Methods:**

We conducted a retrospective review of medical records from patients who underwent postoperative surveillance for primary bone tumors at our institution between 01/06/2016 to 01/09/2023. Diagnostic performance was compared using McNemar's test for paired variables. Sonographic characteristics were analyzed using logistic regression analysis, with statistical significance set at *p* < 0.05.

**Results:**

Comparative analysis revealed no statistically significant differences (*p* > 0.05) in sensitivity, specificity, or accuracy between MRI and US, and the exact values for these parameters are provided in Table 1. Key sonographic features predictive of osteosarcoma recurrence included tumor size and anatomical location. The diagnostic model demonstrated excellent discriminative ability, with an area under the receiver operating characteristic (ROC) curve of 0.973. The diagnostic parameters were as follows: sensitivity (96.6%), specificity (90.9%), accuracy (94.6%), positive predictive value (95.0%), and negative predictive value (93.8%).

**Conclusion:**

The findings from this study support the role of ultrasonography as a valuable tool in tumor surveillance paradigms, providing a scientific rationale for optimizing integrated management strategies in bone oncology.

## Background

Primary malignant bone tumors represent a rare and histologically diverse group of neoplasms originating in bone tissue, encompassing distinct pathological entities such as osteosarcoma (OS), Ewing sarcoma (ES), and chondrosarcoma (CS) [1, 2]. These malignancies demonstrate a higher incidence among adolescents and young adults compared to other cancers, often resulting in significant limb dysfunction and substantial reduction in life expectancy [3]. Recent advances in tumor biology, enhanced preoperative diagnostic imaging, effective neoadjuvant chemotherapy protocols, and refined surgical techniques have established limb-salvage surgery as the preferred approach for local control of aggressive bone tumors [3]. Limb-salvage surgery, defined as a surgical intervention aimed at preserving and restoring bone and joint function following extensive resection of malignant bone tumors in the extremities [4], offers superior functional, psychological, and cosmetic outcomes compared to amputation. However, it carries comparable risks of metastasis and local recurrence (LR) as amputation procedures. Notably, LR serves as a significant prognostic indicator for reduced survival in patients with operable primary osteosarcoma [5], with recurrent lesions exceeding 5 cm and concurrent metastases representing independent predictors of poor prognosis [6]. Early detection of LR through vigilant surveillance may theoretically improve survival outcomes in patients with primary bone tumors [7].

Imaging plays a crucial role in postoperative follow-up; however, there remains a lack of consensus regarding the optimal follow-up protocol after surgery [8, 9]. Each imaging modality possesses its own strengths and limitations. For instance, MRI excels in soft tissue imaging and offers valuable insights for tumor detection, characterization, staging, and post-treatment monitoring [10]. Nevertheless, its imaging quality can be significantly compromised by artifacts caused by metallic prostheses. While ultrasound (US) has inherent limitations in evaluating bone tumors, it demonstrates utility in assessing LR postoperatively and is unaffected by prosthetic artifacts. Currently, there is a paucity of literature on the imaging features of LR following bone tumor surgery. Notably, the ESMO Guidelines Committee's 2021 Clinical Practice Guidelines for the diagnosis, treatment, and follow-up of bone tumors did not establish definitive recommendations for postoperative surveillance of bone tumors, owing to divergent expert opinions and the lack of formal prospective studies [11]. Therefore, this study aims to evaluate the diagnostic performance of MRI and US in detecting local recurrence (LR) of primary malignant bone tumors and to characterize the US imaging features of postoperative soft tissue recurrence in bone tumors.

## Materials and methods

### Study subjects

This study was a retrospective analysis of routinely acquired imaging and clinical data. First, we screened patients from the ultrasound system for postoperative follow-up of bone tumors. The MRI follow-up data and pathological data of the patients were then obtained from the relevant databases. The analysis was performed on the data of patients who were received MRI and US surveillance in Peking University People’s Hospital after surgery for primary bone tumors from 01/06/2016 to 01/09/2023.

### Diagnostic criteria of LR

Two experienced radiologists (Yu Wang and Ping Yu), blinded to pathological data, independently reviewed all images. Discrepancies were resolved through consensus. Multiple recurrences in a single patient were counted as separate cases. Inclusion Criteria for US vs. MRI Comparison: 1) Pathologically confirmed recurrence or non-recurrence; 2) Non-recurrence confirmed by ≥ 6 months of clinical and imaging follow-up; 3) Availability of both US and MRI imaging; 4) Inclusion of diverse bone tumor types (OS, ES, CS). Inclusion Criteria for US Imaging Analysis: 1) Pathologically confirmed recurrence or non-recurrence; 2) Non-recurrence confirmed by ≥ 6 months of clinical and imaging follow-up; 3) Largest lesion selected for patients with multiple lesions; (4) Pathologically confirmed osteosarcoma (OS) cases only. Exclusion criteria: 1) Isolated calcifications; 2) Suspected recurrence without pathological confirmation; 3) Suspected non-recurrence without pathological or ≥ 6-month clinical/imaging follow-up.

### Diagnostic value of MRI and US

MRI and US were used to detect local tumor recurrence after primary bone tumor surgery. The diagnostic performance of each modality was evaluated based on sensitivity, specificity, and accuracy. The following definitions were applied to calculate sensitivity, specificity, and diagnostic accuracy [12]:

1. True positive (TP): Pathologically confirmed LR.
2. False positive (FP): Imaging-suspected LR without pathological confirmation.
3. True negative (TN): Pathologically confirmed absence of LR or ≥ 6 months of recurrence-free clinical follow-up.
4. False negative (FN): Imaging-negative cases with pathologically confirmed LR.

### Sonographic characteristics of LR

Two radiologists—an attending physician (8 years of superficial US experience) and an associate chief physician (14 years of superficial US experience)—conducted double-blind image interpretation. Discrepancies were resolved through consensus. The following sonographic features of LR were evaluated: nodule number (single vs. multiple), maximum lesion diameter, shape (regular vs. irregular), margin (well-defined vs. ill-defined), depth (superficial vs. deep fascia), calcification (present vs. absent), cortical contour (smooth vs. irregular), and vascularity (detectable vs. undetectable).

### Statistical analyses

The McNemar’s Chi-squared test was employed for US-MRI comparisons. Continuous variables (age, lesion diameter) were analyzed using independent sample t-tests, while categorical variables were assessed with McNemar’s test. Statistically significant sonographic features were included as independent variables in binary logistic regression analysis. A regression model was constructed, and diagnostic performance was evaluated using receiver operating characteristic (ROC) curve analysis with area under the curve (AUC) calculation. All analyses were conducted using R (v3.5.1) or SPSS® (v24.0), with *p* < 0.05 considered statistically significant.

## Results

### Results of the US versus MRI comparison study

#### Characteristics of study subjects

Fifty-six cases were involved. The mean age was 20.38 ± 1.60 years, and there were 32 (57.63%) males. Osteosarcoma (*n* = 43) was accounted for 76.79% of all cases, Ewing's sarcoma 12.50% (*n* = 7), and Chondrosarcoma 10.71% (*n* = 6).

#### Diagnostic performance of US and MRI

Regardless of lesion size and position, there was no significant difference (*P* > 0.05) in the sensitivity, specificity and accuracy of the two examination methods (Table 1).

**Table 1:** Diagnostic performance of surveillance US and MRI scans for detection of local recurrent primary bone tumor

Variables	Pathology		Sensitivity	Specificity	Accuracy	PPV	NPV
	Positive	Negative					
**US (*n* = 56)**							
**Positive**	26	2	89.66%	92.59%	91.07%	92.86%	89.29%
**Negative**	3	25					
**MRI (*n* = 56)**							
**Positive**	23	2	79.31%	92.59%	85.71%	92.00%	80.65%
**Negative**	6	25					

*US* Ultrasound, *MRI* Magnetic resonance imaging, *NPV* Negative predictive value, *PPV* Positive predictive value

#### Diagnostic performance and imaging features of the cases with erroneous US/MRI findings

**Fig. 1:**
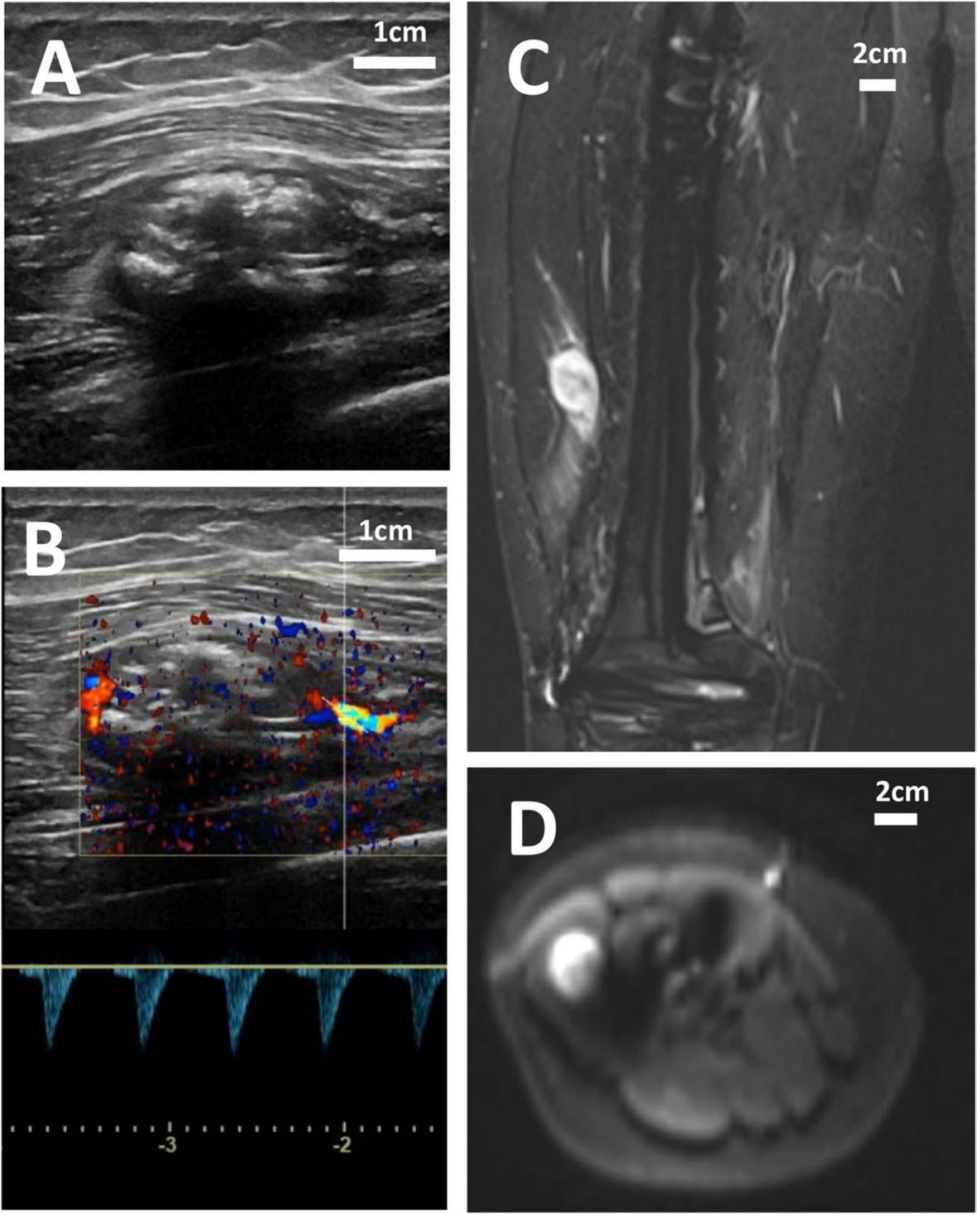
Representative imaging findings of the lesion on US and MRI. **A** Longitudinal US image demonstrating a heterogeneous echogenic mass. **B** Corresponding US image showing intramuscular localization with vascularity and calcifications. **C** MRI STIR sequence revealing perioperative high signal intensity. **D** Diffusion-weighted MRI exhibiting restricted diffusion within the nodule

Figure 1 demonstrates the capability of both US and MRI in detecting LR, though false-positive and false-negative results remain inherent limitations. Two false-positive cases were identified by both modalities, involving lower extremity osteosarcoma. In one case, misdiagnosis likely resulted from sampling error due to limited tissue acquisition via needle biopsy. In the other, diagnostic uncertainty arose from the imaging overlap between inflammatory changes and tumor recurrence.

**Fig. 2:**
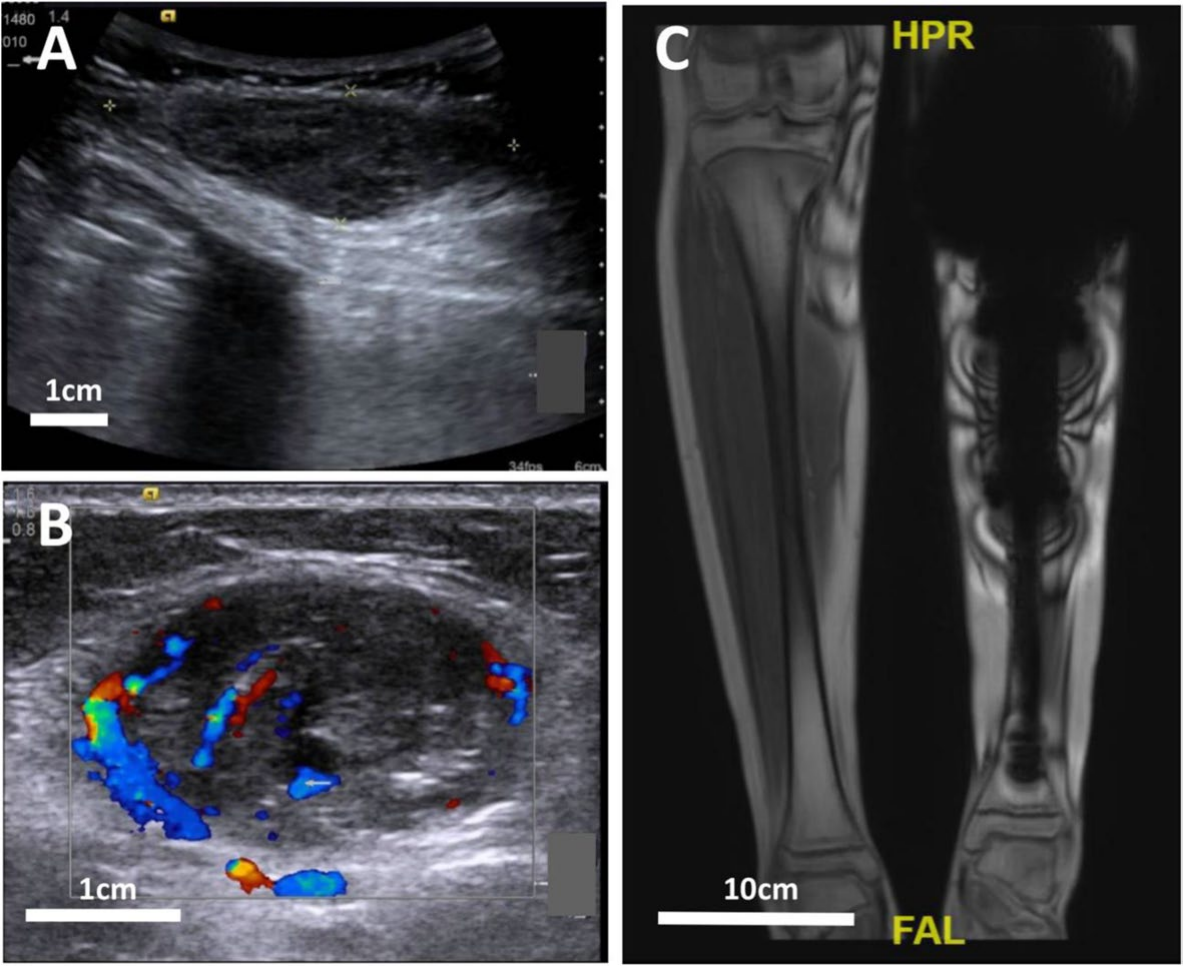
Comparative imaging findings demonstrating lesion visibility on US but not MRI. **A** US image revealing a hypoechoic mass in the left leg's postoperative soft tissue. **B** Additional US image showing a vascularized mass within the lateral gastrocnemius. **C** MRI T1-weighted image obscured by metal prosthesis artifacts, preventing lesion visualization

Three false-negative US findings were identified, all involving thigh osteosarcoma. One case was misdiagnosed due to misinterpretation of recurrence as synovial hyperplasia, while the other two cases resulted from osseous rather than soft tissue recurrence. Six false-positive MRI findings were observed, all in osteosarcoma patients (1 lower extremity, 5 thigh). As illustrated in Fig. 2, all six cases demonstrated significant prosthesis-related artifacts on MRI. Additionally, one case may have been misclassified due to osseous recurrence.

### Results of ultrasonographic feature analysis

#### Results of single-factor analysis

Pathological examination confirmed 59 recurrent and 2 non-recurrent cases, while 31 non-recurrent cases were verified through ≥ 6 months of clinical and imaging follow-up. Compared to the non-LR group, LR cases predominantly exhibited single nodules, larger tumor diameters, and deep fascial involvement (all *P* < 0.05). The non-LR group demonstrated higher rates of calcification and cortical smoothness (both *P* < 0.05). Both groups showed similar characteristics of irregular nodule morphology, well-defined margins, and detectable vascularity, with no significant differences (all *P* > 0.05) (Table 2).

**Table 2:** Clinical data for the patients and single factor analysis of ultrasonographic features

	Definition	Total	no-recurrence	recurrence	*p* Value
**Characteristics**					
Sex					0.898
Women	0	33	15	18	
Man	1	59	26	33	
Age					0.058
Mean ± SD			18.03 ± 1.51	21.83 ± 1.82	
**Imaging features**					
Number of nodules					0.014
Multiple	1	40	18	22	
Single	0	52	15	37	
Size (CM)					< 0.001*
Mean ± SD			1.85 ± 0.14	5.68 ± 0.36	
Shape					0.743
Regular	0	30	13	17	
Irregular	1	62	20	42	
Margin					0.296
Well-defined	1	66	20	46	
Ill-defined	0	26	13	13	
Location					0.039*
Shallow fascia	0	26	25	1	
Deep fascia	1	66	8	58	
Calcification					< 0.001*
Presence	1	24	10	14	
Absence	0	68	23	45	
Cortical smoothness					< 0.001*
Smooth	1	7	2	5	1
Non-smooth	0	92	31	54	
Vascularity					0.665
Detectable	1	55	22	33	
Undetectable	0	37	11	26	

The asterisk designates the statistically significant features if *p* value is less than 0.05

#### Results of multi-factor analysis

Binary logistic regression analysis identified tumor size and growth location as independent predictive factors for distinguishing LR from postoperative changes (Table 3).

**Table 3:** Clinical data for the patients and single factor analysis of ultrasonographic features

Variables	OR	95% C.I		*P* Value
		lower limit	upper limit	
Location	158.231	3.672	6817.813	0.008*
Size	3.547	1.699	7.404	0.001*
Calcification	0.475	0.059	3.837	0.485
Cortical smoothness	2.416	0.002	2624.342	0.805

The asterisk designates the statistically significant features if p value is less than 0.05

*OR* odds ratio, *C.I.* Confidence interval

**Fig. 3:**
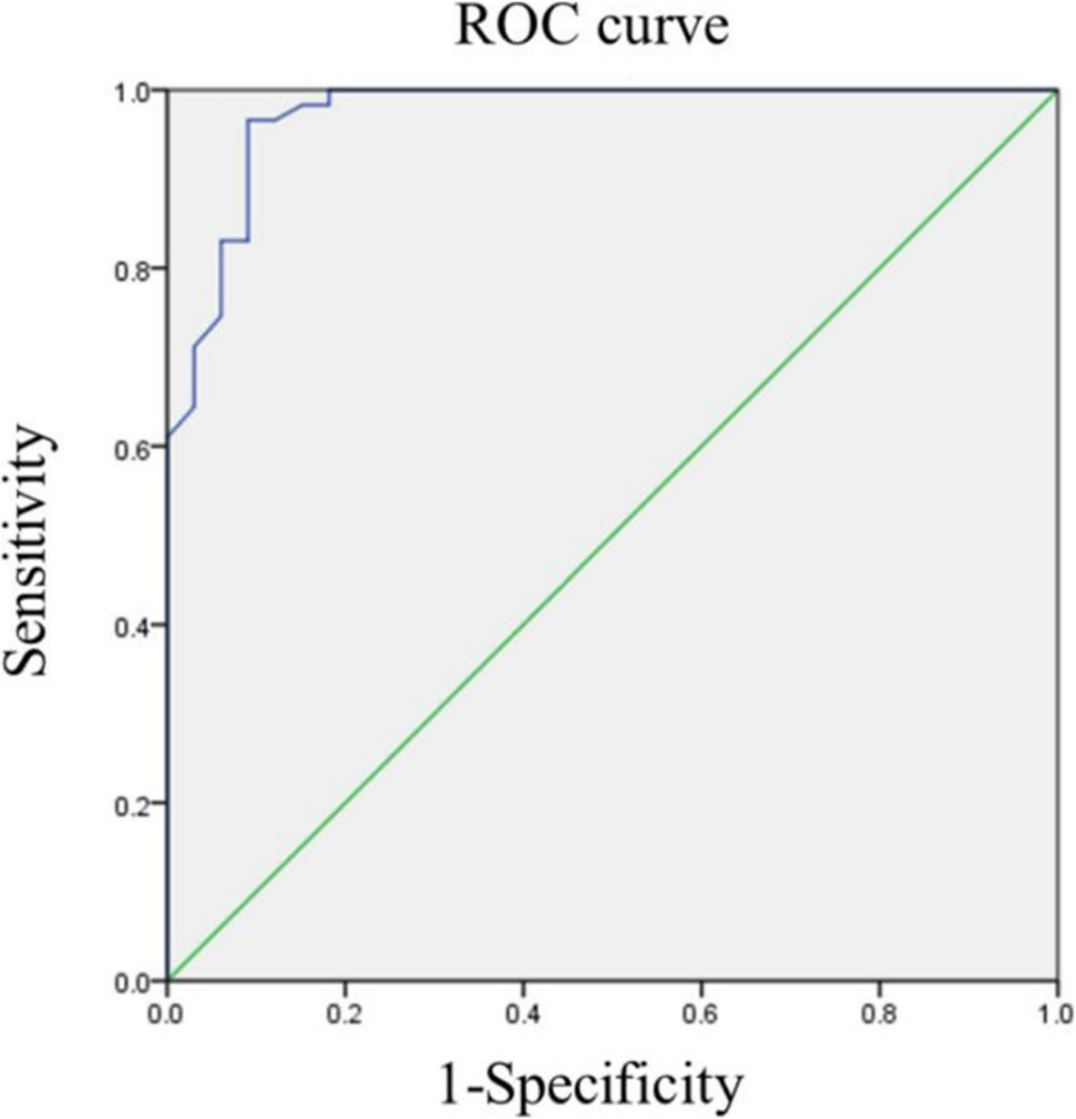
The ROC curve of logistic regression

The diagnostic performance of the logistic regression model for distinguishing LR from non-LR was assessed using ROC curve analysis. The model was defined as: $Logistic\; (Y) = -7.178 +  5.064X_{1}  + 1.266X_{2}$. The area under the ROC curve (AUC) was 0.973, with a maximum Youden's index of 0.875 (Fig. 3). The model demonstrated sensitivity of 96.6%, specificity of 90.9%, and accuracy of 94.6%, with positive and negative predictive values of 95.0% and 93.8%, respectively.

## Discussion

Limb salvage surgery demonstrates comparable postoperative survival rates to radical resection [4], making it the preferred choice for patients with malignant bone tumors [13]. Prosthetic reconstruction remains the most widely utilized and effective limb salvage technique. Recent surveys have shown that the clinical 10-year satisfaction rate of a good quality prosthesis can reach 90% [14]. However, metal implants generate significant artifacts on MRI [15, 16], limiting its utility despite excellent soft tissue contrast for evaluating musculoskeletal structures, neurovascular relationships, and tumor extent [17–19]. As demonstrated in Fig. 2, US enables better lesion visualization compared to MRI in these cases due to its immunity to metallic artifacts.

Akihiko et al. demonstrated a 30% 5-year overall survival rate following osteosarcoma LR, identifying recurrent tumor size as a key prognostic indicator [20]. Early LR detection is therefore critical for improving patient outcomes [12]. However, small LR lesions are often non-palpable and challenging to differentiate from post-radiotherapy fibrosis or scar tissue. These recurrences may occur at various sites, including the primary tumor location, resection margins, or periprosthetic regions. Conventional radiographs and bone scans frequently fail to detect soft tissue recurrences lacking mineralized osteoid [21]. Our previous work established US as a non-invasive first-line imaging modality for detecting LR in primary bone tumors: the sensitivity and accuracy of US was higher than that of x-ray, and there is demonstrated no statistically significant differences in the sensitivity, specificity and accuracy among US, CT and 99 mTc-MDP bone scan [12]. The current study further demonstrates comparable efficacy between US and MRI for postoperative surveillance. Notably, this investigation represents one of the first comprehensive analyses of sonographic predictors for postoperative osteosarcoma LR. Logistic regression identified lesion size and deep fascial location as significant diagnostic indicators. Future research should use advanced ultrasonographic techniques to enhance postoperative surveillance strategies for bone tumors, addressing current limitations and optimizing diagnostic accuracy in clinical practice.

Several limitations should be acknowledged in this study. First, a sample size of less than 100 cases is a relatively small cohort that makes it difficult to draw definitive conclusions. Second, the retrospective nature of this study limits the generalizability of these findings, and a prospective study is needed in the future. Third, Interobserver variability in image interpretation among radiologists could affect diagnostic consistency as ultrasound interpretation depends on the operator-dependent variability. Finally, while MRI provides comprehensive evaluation of both osseous and soft tissue structures throughout the entire limb, US is limited to targeted soft tissue assessment. Our study focused specifically on comparing these modalities for detecting soft tissue recurrence following bone tumor surgery, rather than evaluating their complete diagnostic capabilities.

## Conclusions

While MRI demonstrates high sensitivity for musculoskeletal evaluation, its utility in postoperative surveillance of prosthetic reconstruction is significantly limited by metal-induced artifacts that compromise soft tissue assessment. In such cases with metal artifacts, US is complementary to MRI. Our logistic regression analysis identified lesion size and deep fascial location as key sonographic predictors of osteosarcoma recurrence. The robust evidence derived from this investigation underscores the pivotal role of ultrasonography in contemporary tumor surveillance paradigms, providing a scientific rationale for optimizing integrated management strategies in bone oncology.

## Data Availability

The datasets used and/or analyzed during the current study are available from the corresponding author on reasonable request.

## Abbreviations

US: Ultrasound
MRI: Magnetic resonance imaging
OS: Osteosarcoma
ES: Ewing sarcoma
CS: Chondrosarcoma
PPV: Positive predictive value
NPV: Negative predictive value
TP: True positive
FP: False positive
TN: True negative
FN: False negative
LR: Local soft tissue recurrence

## References

[1] Choi JH, Ro JY. The 2020 WHO classification of tumors of bone: an updated review. Adv Anat Pathol. 2021;28(3):119–38. 33480599 10.1097/PAP.0000000000000293

[2] Folkert IW, Devalaraja S, Linette GP, Weber K, Haldar M. Primary bone tumors: challenges and opportunities for CAR-T therapies. J Bone Miner Res. 2019;34(10):1780–8. 31441962 10.1002/jbmr.3852

[3] Tan TJ, Aljefri AM, Clarkson PW, Masri BA, Ouellette HA, Munk PL, Mallinson PI. Imaging of limb salvage surgery and pelvic reconstruction following resection of malignant bone tumours. Eur J Radiol. 2015;84(9):1782–90. 26104572 10.1016/j.ejrad.2015.06.002

[4] Xu M, Wang Z, Yu XC, Lin JH, Hu YC. Guideline for limb-salvage treatment of osteosarcoma. Orthop Surg. 2020;12(4):1021–9. 32633103 10.1111/os.12702 PMC7454155

[5] Weeden S, Grimer RJ, Cannon SR, Taminiau AH, Uscinska BM. The effect of local recurrence on survival in resected osteosarcoma. Eur J Cancer. 2001;37(1):39–46. 11165128 10.1016/s0959-8049(00)00362-2

[6] Li X, Moretti VM, Ashana AO, Lackman RD. Impact of close surgical margin on local recurrence and survival in osteosarcoma. Int Orthop. 2012;36(1):131–7. 21404025 10.1007/s00264-011-1230-x PMC3251690

[7] Zhang X, Guan Z. PET/CT in the diagnosis and prognosis of osteosarcoma. Front Biosci (Landmark edition). 2018;23:2157–65. 10.2741/4696 29772552

[8] Brenner W, Bohuslavizki KH, Eary JF. PET imaging of osteosarcoma. J Nucl Med. 2003;44(6):930–42. 12791822

[9] Fletcher BD. Imaging pediatric bone sarcomas. Diagnosis and treatment-related issues. Radiol Clin North Am. 1997;35(6):1477–94. 9374999

[10] Inarejos Clemente EJ, Navarro OM, Navallas M, Ladera E, Torner F, Sunol M, Garraus M, March JC, Barber I. Multiparametric MRI evaluation of bone sarcomas in children. Insights Imaging. 2022;13(1):33. 35229206 10.1186/s13244-022-01177-9 PMC8885969

[11] Strauss SJ, Frezza AM, Abecassis N, Bajpai J, Bauer S, Biagini R, Bielack S, Blay JY, Bolle S, Bonvalot S, et al. Bone sarcomas: ESMO-EURACAN-GENTURIS-ERN PaedCan Clinical Practice Guideline for diagnosis, treatment and follow-up. Annals of oncology : official journal of the European Society for Medical Oncology. 2021;32(12):1520–36. 34500044 10.1016/j.annonc.2021.08.1995

[12] Wang Y, Yu P, Liu F, Wang Y, Zhu J. Clinical value of ultrasound for the evaluation of local recurrence of primary bone tumors. Front Oncol. 2022;12: 902317. 36185277 10.3389/fonc.2022.902317 PMC9520522

[13] Han G, Bi WZ, Xu M, Jia JP, Wang Y. Amputation versus limb-salvage surgery in patients with osteosarcoma: a meta-analysis. World J Surg. 2016;40(8):2016–27. 27116252 10.1007/s00268-016-3500-7

[14] Walker MJ. On Replacement Body Parts. Journal of bioethical inquiry. 2019;16(1):61–73. 30565032 10.1007/s11673-018-9889-y

[15] Hargreaves BA, Worters PW, Pauly KB, Pauly JM, Koch KM, Gold GE. Metal-induced artifacts in MRI. AJR Am J Roentgenol. 2011;197(3):547–55. 21862795 10.2214/AJR.11.7364 PMC5562503

[16] Boas FE, Fleischmann D. Evaluation of two iterative techniques for reducing metal artifacts in computed tomography. Radiology. 2011;259(3):894–902. 21357521 10.1148/radiol.11101782

[17] Chang G, Boone S, Martel D, Rajapakse CS, Hallyburton RS, Valko M, Honig S, Regatte RR. MRI assessment of bone structure and microarchitecture. Journal of magnetic resonance imaging : JMRI. 2017;46(2):323–37. 28165650 10.1002/jmri.25647 PMC5690546

[18] Xu H, Othman SF, Magin RL. Monitoring tissue engineering using magnetic resonance imaging. J Biosci Bioeng. 2008;106(6):515–27. 19134545 10.1263/jbb.106.515

[19] Hirschmann A, van Praag VM, Haas RL, van de Sande MAJ, Bloem JL. Can we use MRI to detect clinically silent recurrent soft-tissue sarcoma? Eur Radiol. 2020;30(9):4724–33. 32314057 10.1007/s00330-020-06810-z

[20] Takeuchi A, Lewis VO, Satcher RL, Moon BS, Lin PP. What are the factors that affect survival and relapse after local recurrence of osteosarcoma? Clin Orthop Relat Res. 2014;472(10):3188–95. 24980644 10.1007/s11999-014-3759-7 PMC4160478

[21] Eftekhari F. Imaging assessment of osteosarcoma in childhood and adolescence: diagnosis, staging, and evaluating response to chemotherapy. Cancer Treat Res. 2009;152:33–62. 20213385 10.1007/978-1-4419-0284-9_3

